# Microplastics: Omnipresent and an ongoing challenge for medical science

**DOI:** 10.1007/s00508-024-02375-9

**Published:** 2024-05-21

**Authors:** Hans-Peter Hutter, Lisbeth Weitensfelder, Michael Poteser

**Affiliations:** Department of Environmental Health, Center for Public Health, Medical University of Vienna, Vienna, Austria

**Keywords:** Microplastics, Nanoplastics, Health risks

## Abstract

Micro- and nanoplastics are omnipresent not only in the environment, but have also been detected in human body fluids and tissue. The subsequent commentary provides a perspective about potential risks for human health as well as resulting challenges for medical science.

Microplastics (particles < 5 mm) and nanoplastics (particles < 0.1 µM) are increasingly being detected not only ubiquitously in the environment, but also in animal and human tissues and body fluids.

Environmental health focuses on the health effects of environmental factors which—due to the ever increasing footprint of human activities—are changing the state of the natural environment. New, mostly industrially used, substances are often being associated with human health risks. As we have seen with industrial chemicals, pesticides, heavy metals, particulate matter as well as physical factors such as noise and radiation, it is a question of the availability of scientific evidence as to if and how much the health impacts can ultimately be reduced or avoided.

Biomonitoring studies have shown that certain substance classes of environmental pollutants can already be suspected of posing a health risk due to their adverse outcome pathways (AOPs) [1]. However, there are industrial environmental pollutants of which health effects in humans are difficult to predict due to their properties.

These include, for example, the so-called “Forever Chemicals”, PFAS (per- and polyfluorinated alkyl compounds), which are difficult to break down due to their physico-chemical inertness and which accumulate in the environment as well as in living organisms. For a large part of the European population, exposure to PFAS is now considered to be too high [2], especially the increased concentrations in European teenagers [3]. Despite this omnipresence, research is only just beginning to understand how these chemicals can affect or harm human health. This also applies to an even more persistent group of substances that cannot be ignored in view of their mass in the (immediate) human environment: Microplastics.

Microplastics are inert regarding their chemical properties and therefore initially caused little concern. However, very small microplastic particles, called nanoplastic (< 1 µM), are increasingly suspected of damaging the lipid membranes of cells as well as producing oxidative stress [4–6].

A further potential risk arises from chemicals [7] or pathogens [8, 9] that can be transported on or in these particles. Numerous additives are added to plastics, such as plasticisers, flame retardants or colourants. These chemical substances are usually very weakly bound and can therefore be easily released from the plastic. Many of these chemicals can have effects on the endocrine system (endocrine disruptors), others have an effect on the nervous or reproductive system and some are considered carcinogenic. Alongside these chemical properties, there is another dimension of plastic particles to consider. Depending on their size and shape, they can have very different effects on the environment and organisms. The smaller the plastic particle, the higher the probability that it can penetrate into cells. At the same time, the larger its surface area the more pollutants can be adsorbed and accumulate.

Environmental toxicological experience with persistent chemicals has taught that we must not be fooled by the inertness of persistent environmental pollutants. On the contrary, the fact that no clear reaction pathways (or AOPs) are initially foreseeable could imply that completely unexpected effects may occur in various biological systems including the human body. Such effect can only be uncovered by unbiased, open-ended broad research.

A recently published study found for the first time that microplastics present in atheroma obtained from asymptomatic carotid plaques increase the hazard for myocardial infarction, stroke or all-cause death about 4.5-fold [10]. Thus there is now evidence that microplastics indeed pose a human health risk. This finding is alarming and will not be the final surprise.

## Microplastic iceberg

Considering this and other recent findings related to microplastics, we are probably not going too far in predicting that mankind is facing a major health problem here. The studies available to date may only show the tip of the “Microplastic Iceberg”. Meanwhile, more and more plastic particles are entering our environment.

A distinction must be made between primary microplastics, i.e. plastic particles produced specifically in microscopic size for cosmetics, detergents and cleaning agents, and secondary microplastics. The latter is mainly produced by the decomposition and fragmentation of larger plastics, from: packaging material, tire abrasion from vehicles, and disposable medical products. Millions of tons of plastics are released into the environment every year. According to estimates more than eight billion tons of plastic have been produced worldwide to date. Only a comparatively small proportion is recycled or incinerated; almost 80% is found in landfill sites, terrestrial ecosystems or the world’s oceans [11]. A not insignificant proportion of the (disposable) plastic products and waste is turned into microplastics.

We ingest the largest proportion of microplastics via food and water chains. The main sources are fish and other marine animals, which have already ingested plastic particles themselves [12]. Terrestrial and aquatic ecosystems, especially the world’s oceans, are “reservoirs” for plastic waste. House dust also appears to be a relevant source of ingestion [13]: Toddlers of crawling age in particular can ingest microplastics through hand to mouth contact. Last but not least, microplastics are absorbed via the respiratory tract [14].

In the case of larger particles, it can be assumed that they are excreted via the digestive tract. However, with smaller particles in the nanometer range there is a risk that they will be deposited in the intestinal tissue and also in the respiratory tract or lung tissue—as happens with soot particles. Fine particles enter the bloodstream, where they can not only be deposited in the blood vessels, as has recently been shown, but can be distributed everywhere in the body and even enter the amniotic fluid via the placenta as we have recently shown [15]. In animal studies it was shown that ingested micro- and nanoplastic particles can not only be found in numerous organs (e.g. liver, heart, kidneys), but can even cross the blood-brain barrier [12].

As mentioned above, not only the particles themselves are a concern but also the adsorbed chemicals (and microorganisms) an aspect that has only begun to be explored and that points to an even more pronounced problem for ecosystems and human health [16].

## Risk assessment

The assessment of microplastics in biological tissue is challenging due to the difficulties both in detecting them and in determining their composition. As a result, important information for a risk assessment is missing in the majority of cases. Due to their nature as rather inert carbon compounds, the tiny microplastics are hidden from common detection methods in bodily fluids amidst a myriad of naturally occurring particles. We were participating in the development of a method for the simultaneous identification and detection of nanoparticles in amniotic fluid [15]. Similar challenges have to be overcome for other biological matrixes.

It is not yet possible to reliably assess the exact risk for humans posed by microplastics. Toxicokinetic studies have so far been conducted on whether and how uptake into organisms and cells takes place [17]. There is some consensus that very small plastic particles are actively or passively taken up by cells depending on their features. The cells then attempt to degrade the particles biochemically, but this is hindered due to the chemical persistence of plastics. This can lead to an increase in reactive oxygen species in the cells and damage to intracellular compartments [18].

Another major area of research is the role of microplastics in immune responses. Since these small particles seem to be biologically interpreted as foreign, they can trigger inflammatory reactions [19, 20].

The challenge for science is twofold: New innovative detection methods in biological tissue and body-fluids for micro- and nanoplastics must be developed and basic toxicological studies, clinical and epidemiological research need to address the full range of effects that these particles have in humans and other species. It is pleasing that research in Austria has recognized this responsibility and accepted the challenge.

## Outlook

Microplastics are not only a matter of concern for human health, but also for ecosystems—which in turn interact with human health. Microplastics can occur as a degredation product of macroplastics. Considering the enormous amount of plastic that is and has been produced, the presence of microplastics in the environment will not decline in the following decades, if not centuries. Further efforts to reduce plastics use and to avoid plastic waste are necessary to decrease the burden of microplastics. In addition, methods to capture and reduce microplastic having already entered the biosphere and especially the water systems must be developed and applied to protect nature and human health.

The presence of plastics in geological deposits is cited as one of the clear geological characteristics for the present era and it may, for this and other reasons, be justified to speak of an ‘Anthropocene’. It can also be seen as a global responsibility to develop strategies for the protection of future generations from the consequences of our remains, so that plastics and their degradation products do not stay forever.

## Data Availability

There are no data for this paper.
